# Machine Learning Bolsters Evidence That D1, Nef, and Tat Influence HIV Reservoir Dynamics

**DOI:** 10.20411/pai.v8i2.621

**Published:** 2024-01-23

**Authors:** LaMont Cannon, Sophia Fehrman, Marilia Pinzone, Sam Weissman, Una O'Doherty

**Affiliations:** 1 Center for Biological Data Science, Virginia Commonwealth University, Richmond, Virginia; 2 Department of Pathology and Laboratory Medicine, University of Pennsylvania, Philadelphia, Pennsylvania

**Keywords:** HIV Reservoir, Machine Learning, NFL Sequencing

## Abstract

**Background:**

The primary hurdle to curing HIV is due to the establishment of a reservoir early in infection. In an effort to find new treatment strategies, we and others have focused on understanding the selection pressures exerted on the reservoir by studying how proviral sequences change over time.

**Methods:**

To gain insights into the dynamics of the HIV reservoir we analyzed longitudinal near full-length sequences from 7 people living with HIV between 1 and 20 years following the initiation of antiretroviral treatment. We used this data to employ Bayesian mixed effects models to characterize the decay of the reservoir using single-phase and multiphasic decay models based on near full-length sequencing. In addition, we developed a machine-learning approach utilizing logistic regression to identify elements within the HIV genome most associated with proviral decay and persistence. By systematically analyzing proviruses that are deleted for a specific element, we gain insights into their role in reservoir contraction and expansion.

**Results:**

Our analyses indicate that biphasic decay models of intact reservoir dynamics were better than single-phase models with a stronger statistical fit. Based on the biphasic decay pattern of the intact reservoir, we estimated the half-lives of the first and second phases of decay to be 18.2 (17.3 to 19.2, 95%CI) and 433 (227 to 6400, 95%CI) months, respectively.

In contrast, the dynamics of defective proviruses differed favoring neither model definitively, with an estimated half-life of 87.3 (78.1 to 98.8, 95% CI) months during the first phase of the biphasic model. Machine-learning analysis of HIV genomes at the nucleotide level revealed that the presence of the splice donor site D1 was the principal genomic element associated with contraction. This role of D1 was then validated in an *in vitro* system. Using the same approach, we additionally found supporting evidence that HIV *nef* may confer a protective advantage for latently infected T cells while *tat* was associated with clonal expansion.

**Conclusions:**

The nature of intact reservoir decay suggests that the long-lived HIV reservoir contains at least 2 distinct compartments. The first compartment decays faster than the second compartment. Our machine-learning analysis of HIV proviral sequences reveals specific genomic elements are associated with contraction while others are associated with persistence and expansion. Together, these opposing forces shape the reservoir over time.

## INTRODUCTION

The treatment of HIV-infected individuals with antiretroviral therapy (ART) results in a rapid decline in viremia and a dramatically improved lifespan [1]. While ART effectively prevents new infections, its discovery revealed a reservoir of latently infected cells that persists despite long-term ART. When ART is stopped, it is postulated that quiescent resting T cells become activated and initiate new rounds of infection leading to recurrent viremia. This hypothesis is based on evidence that stimulating resting CD4+ T cells *ex vivo* leads to viral spread. The Quantitative Viral Outgrowth Assay (QVOA) is based on this finding and provides an estimate of reservoir size by stimulating T cells at limiting dilution and counting the frequency of viral outgrowth [2–6]. The outgrowth assay has the advantage of excluding defective proviruses and provides an average half-life of 44 months for the HIV reservoir [6, 7].

Reservoir kinetics appear to be multiphasic as suggested by highly sensitive viral RNA measures [8]. These studies suggest there are at least 4 phases of reservoir dynamics. The first and second phase are likely dominated by decay of more activated infected cells with half-lives of 1 to 2 days and 2 to 3 weeks [9]. The third and fourth phases are more difficult to measure by RNA and are thought to represent kinetics of latently infected T cells [8, 10]. Estimates of the third and fourth phases have also been made using both Intact Proviral DNA Assay (IPDA) and QVOA [11–13]. These measures suggest there is significant heterogeneity in the third and fourth phases of reservoir dynamics with a significant portion of patients showing expansion of the reservoir during the fourth phase. Understanding the multiphasic nature of HIV reservoir dynamics and heterogeneity among individuals will be essential to HIV cure.

The forces that determine reservoir dynamics, especially during the later phases, remain unclear. If the reservoir were transcriptionally silent, then only external forces such as antigen stimulation and homeostatic signals would drive reservoir dynamics. However, there is an increasing appreciation that at least a portion of the HIV reservoir is not transcriptionally silent, and thus it is possible that proviral expression may contribute to reservoir dynamics even during the fourth phase. To further probe the complexity of reservoir dynamics, we and others examined reservoir dynamics by analyzing changes in HIV sequences over time [14–16]. Intact proviruses were identified bioinformatically, and dynamics were studied utilizing a large number of time points. While these estimates of reservoir decay by sequencing are consistent with previous QVOA estimates [14–16], they also revealed additional insights into reservoir dynamics. In particular, it appears that reservoir contraction is countered by episodes of clonal expansion [14]. These forces of contraction and expansion continue beyond the third phase [11, 13, 14]. To dissect the role of proviral genetic elements on reservoir contraction and expansion, we focused our analysis on defective proviruses. Inherent in this approach is the assumption that the proviruses are transcriptionally active, and this expression contributes to both contraction and expansion forces. This complements other work that focuses on the role of antigen and homeostasis on reservoir dynamics [17, 18].

By analyzing changes in sequences of defective proviruses over time, we found evidence of external selection forces. The proportion of those proviruses with 3’ deletions (D1+ with a complete ORF) decay relative to the other proviruses while those with 5’ deletions (D1-D4+) increase relative to the other proviruses. We hypothesized that the 3’ deleted proviruses might decay faster because they expressed HIV proteins more efficiently. Consistent with our smaller study, Peluso et al examined proviral dynamics in a larger cohort using the IPDA assay, also revealing that proviruses with D1 decay faster than those without [12]. Taken together, IPDA and sequencing approaches reveal that intact proviruses decay faster than defective ones, consistent with the original decay estimates by QVOA compared to slower decay of total DNA [6, 19].

In this manuscript, we analyzed intact and defective proviral dynamics. We first analyzed near full-length sequences from a larger cohort to fit mathematical models of reservoir dynamics for intact and defective proviruses separately. The sequences were then utilized to develop a machine-learning algorithm to gain more insight into the dynamics. To increase our resolution, we dissected the HIV genome into precise elements. We then systematically evaluated the contribution of each element to reservoir contraction and expansion. We asked if specific elements within the HIV genome are associated with proviral decay as evidenced by their presence in the genomes that decay quickly. Likewise, we asked if other elements within the HIV genome are associated with proviral persistence. Simultaneously, we also investigated which elements predict proviral expansion by examining elements associated with proviral clones.

## METHODS

### Definitions

A **proviral clone** is defined as 2 or more identical proviral sequences. The majority of repeated sequences in our study also contain a deletion with a unique junction. A provirus with an identical nucleotide sequence and an identical deletion likely represents a true proviral clone; however, without the integration site, it is remotely possible that a fraction of our proviral clones is not actually clonal. However, this is more likely an issue for our intact proviral sequences that lack a deletion.

We define the **intact reservoir** as those proviruses that appear to be intact bioinformatically. Specifically, an intact HIV provirus is defined as a provirus containing 9 ORFs, the 4 stem loops that comprise the psi packaging site, the rev response element (RRE) as well as critical splice sites including D1, D4, and A4a/b/c, A5, and A7. This serves as a surrogate of the rebound competent reservoir. The criteria for the 33 genetic elements defined by nucleotide sequence are listed in Supplementary Table 1 and are adapted from our previous work [14].

**Table 1.** Model Fit Data

Intact	AICc		RMSE	
	All Data	Clones Removed	All Data	Clones Removed
**Single Phase**	992.21	692.09	47.31	32.35
**Biphasic**	851.84	676.16	27.50	16.96

Defective	AICc		RMSE	
	All Data	Clones Removed	All Data	Clones Removed
**Single Phase**	534.65	517.99	357.18	268.65
**Biphasic**	549.34	535.28	353.41	269.86

**Figure 1.**
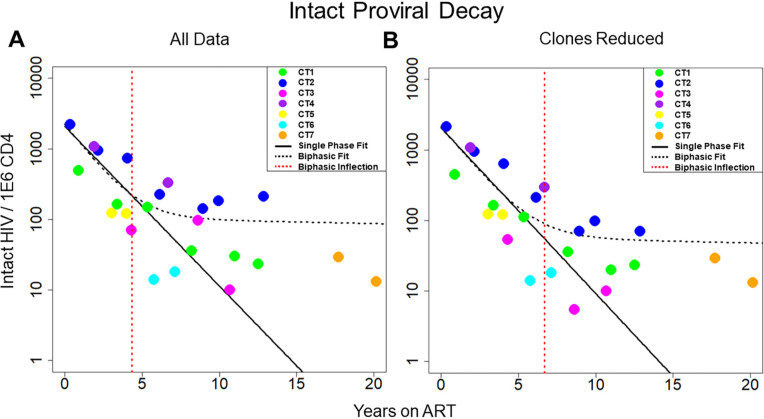
**Decay of intact HIV**. Concentration of intact HIV provirus per million CD4 plotted at all time points for the 7 individuals in the study. Thick black line represents the best fit to a single-phase decay model. Dotted black line represents best fit to a biphasic decay model. The dotted red line represents the inflection point of the biphasic model. A) Best fit of the 2 models with all data included in the analysis. B) Best fit of the 2 models with clones removed. The fit of the 2 models is drastically different both with all data included and with clones removed, with the biphasic model providing a much better fit to the data. CT stands for chronically treated. In other words, an individual who was treated during the chronic phase of infection.

**Figure 2.**
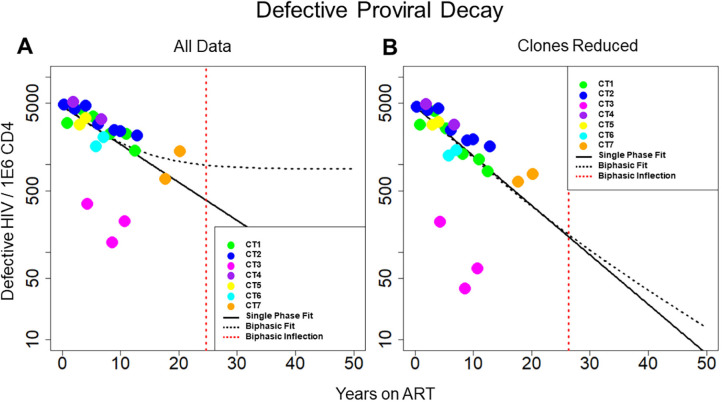
**Decay of defective HIV**. Concentration of defective HIV proviruses per million CD4 plotted at all time points for the 7 individuals in the study. Thick black line represents the best fit to the single-phase model. Dotted black line represents the best fit to the biphasic decay model. The dotted red line represents the inflection point of the biphasic model. A) Best fit of the 2 models with all data included in the analysis. B) Best fit of the 2 models with clones removed. With all of the data included, the biphasic model provides a slightly better fit to the data; however, with the clones removed, the model fits are more similar between the single-phase and biphasic models.

**Reducing clones** (Figures 1 and 2). We reduce clones by representing a repeated proviral sequence only once the first time it appears.

**Figure 3.**
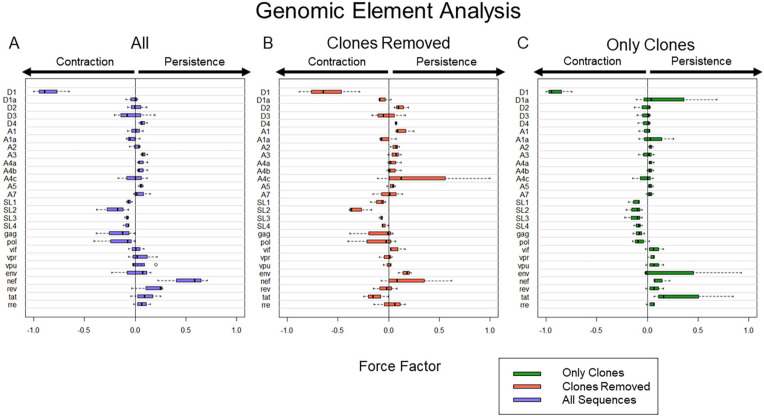
**Computational expression of HIV elements and their association with proviral persistence vs decay.** A) Frequency of HIV elements with all proviral data included. B) Frequency of HIV elements with all definitive clonal population removed. C) Frequency of HIV element using proviral sequences from definitive clonal populations. To present the data more clearly, Force Factors for only 28 of the 33 elements considered in our analysis are shown. Env loops V1-V5 were excluded as none of them had a large individual contribution to either persistence or decay. Notably, the upper and lower 5% of sequences associated with the most decay and most persistence are different in A, B, and C. Thus, A is not an average of B and C.

**Removing clones** (Figure 3). We remove clones by removing all repeated sequences from the analysis.

**Clones only** (Figure 3). We analyze the repeated sequences over time.

### Patients and Sequences

HIV sequences were acquired from 7 individuals living with HIV and receiving chronic treatment, using near full-length PCR at limiting dilution followed by sequencing on an Illumina platform as previously described [14]. All participants were receiving ART during the entire length of the sample collection period. Clinical characteristics of the cohort are provided in Supplementary Table 2. Sequences were analyzed in R using an in-house HIV genome analysis software. The number of intact and defective sequences analyzed per patient at every timepoint is provided in Supplementary Table 3.

**Table 2.** Log Bayes Factors

Log Bayes Factors			
		All Data	Clones Removed
		Single Phase	Single Phase
**Intact**	**Biphasic**	35.603	8.582
**Defective**	**Biphasic**	1.936	1.370

### Model

We used Bayesian Mixed Modeling methods to compare 2 different models.


(1)

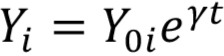



(2)

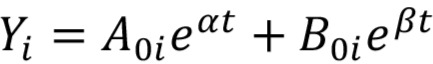


Our first model, defined in equation (1), represented the first order decay of the HIV reservoir for each of the i = {1,2,3,4,5,6,7} participants in our study. A single phase of decay was applied using a fixed decay rate γ for all participants. We allowed for random effects due to different initial reservoir concentrations Y_oi_ = {Y_o1_,…, Y_o7_} for all 7 individuals in the study. The second model also incorporated first order decay dynamics but included biphasic decay. Identified by equation (2), the biphasic model was segmented into 2 compartments and allowed for random effects from different initial reservoir concentrations, where A_oi_ = {A_o1_,…, A_o7_} represented the initial concentration of the first compartment and B_oi_ = {B_o1_,…, B_o7_} represented the initial concentration in the second compartment for each of the 7 participants. Decay rates α and β represented the decay rate of the first and second compartments, respectively, and were fixed for all 7.

### Bayesian Analysis

Models were fit to experimental data using Bayesian Markov Chain Monte Carlo (MCMC) techniques using the Stan statistical inference tool. The MCMC was accomplished using 4 parallel chains with 50,000 iterations per chain and a burn-in process utilizing 10,000 iterations. The MCMC results were only accepted if all 4 chains converged. Computational analysis was done in the R programming environment using R (version 4.0.5), rstan (version 2.21.2), and rstantools (version 2.1.1) [20]. Models were then compared and validated using Widely Applicable Information Criteria (WAIC) and Leave One Out Cross-Validation (loo R package version 2.2.1) [21]. Bayes factors for model comparison were determined using bridgesampling methods to estimate marginal likelihood using R packages brms (version 2.15.0) and bridgesampling (version 1.1-2) [22, 23].

### Computational Expression Analysis

We developed a computational method to analyze the impact of the various elements in the HIV genome on overall proviral dynamics. Because multiple genetic elements in the HIV provirus are required to express most of the HIV proteins, it follows that we should evaluate the genetic elements in combination. For example, every HIV protein, except for gag and pol, requires functional splice sites to generate mRNA capable of making a functional HIV protein [24, 25]. Thus, our method employed a machine-learning strategy to quantify the effect of each genomic element on proviral decay in an unbiased manner using combinatorics. The algorithm is described in greater detail in Supplementary Figure 1. Briefly, the algorithm performs a logistic regression on combinations of elements in the genome at multiple time points. Each combination of elements is then associated with a logistic regression rate coefficient. We chose to study combinations of 4 elements as approximately 93% of our sequences contained at least 4 elements. Additionally, we performed a stepwise regression analysis, successively adding one element at a time and studying its effect on the dynamics. In doing so, we found that most interactions were limited to 2 or 3 elements, thus we restricted the algorithm to 4 element combinations from a total of 33 elements. This led to the analysis 40,920 different combinations or C(33, 4), where C(A,B) represents the binomial coefficient calculation of the number of ways to choose B unordered elements from a set of A elements by combinatorics. The elements considered were 14 splice donor and acceptor sites (D1, D1a, D2, D3, D4, A1, A1a, A2, A3, A4a, A4b, A4c, A5, and A7), the psi packaging site stem loops (S1, S2, S3, and S4), 5 Env variable loops (V1, V2, V3, V4, and V5), 9 ORFs (*gag, pol, vif, vpr, vpu, env, nef, rev,* and *tat*), and the *rev* response element (rre). In Figure 3, the data for V1-V5 are not shown. The regression coefficients associated with each combination of elements serve as surrogate measures of proviral contraction (lowest values) or persistence (highest values). We ranked each combination in order of their regression coefficients. We analyzed the lower 5% of combinations, which are associated with proviral decay, for the presence of each element. Therefore, we examined the smallest coefficients, ie, the bottom 5% of all combinations (0.05 * 40,920 = 2,046). Similarly, we then analyzed the upper 5% of combinations, associated with proviral persistence, for the presence of each element. Choosing a 5% cutoff allows us to minimize noise while still maintaining a strong signal. The relative prevalence of each element is thus representative of its role in either proviral contraction or persistence. We then subtracted the number of occurrences of each element in the upper 5% from the number of occurrences of the same element in the lower 5%. Under the null hypothesis, if a given element is not associated with either decay or persistence, then we would expect its prevalence in the upper 5% to be equal to its prevalence in the lower 5%. We predict that an element is associated with contraction if it appeared more in the lower 5%, thus the difference would be negative; for example, D1_upper_ – D1_lower_ was negative. Conversely, if the element appeared more in the upper 5%, we predict an association with persistence; similarly, *nef*_upper_ – *nef*_lower_ was positive. We normalized our signal by dividing these differences by the total amount of possible times an element could appear in either group (2,046). This creates a number ranging from -1 to 1, which we refer to as the genomic force factor. If a given element is found exclusively in the lower group and in every combination, then this factor would be -1. Conversely if an element is found exclusively in the upper group and in every combination, it would have a factor of 1. A factor of 1 is the highest possible association with persistence, -1 is the highest possible association with decay, and 0 is an association with neither. We performed this algorithm on the 3 participants in our cohort whom we had sequenced at more than 2 time points. Supplementary Figure 1 provides a visual representation of our analysis.

### Force Factor P Values by Permutation

To determine the statistical significance of each of the force factors we calculated each *P* value computationally using a permutation method. We first simulated 1,000 sequences at each time point. Each sequence contained a random permutation of the presence or absence of each element. We then calculated the regression rate coefficient for each of the 40,920 different 4 element combinations and used them to calculate the force factor for the first element. We then repeated these steps 100,000 times using a different random permutation of sequences each time. This allowed us to build a null distribution for the force factor. This distribution is shown in Supplementary Figure 2. *P* values for each element were estimated by calculating the proportion of permuted force factors in the null distribution that were equal or more extreme than the observed force factor for a given element. We then performed the Benjamin Hochberg procedure to calculate adjusted *P* values to correct for multiple comparisons.

## RESULTS

### Modeling Intact HIV Reservoir Dynamics

We sought to measure the decay of the intact reservoir by near full-length sequencing and compared the results to previous methods. To estimate decay characteristics of the HIV reservoir, the data were fit to both single-phase and biphasic decay mixed effects regression models (Figure 1A). Both models were fit using Bayesian Markov Chain Monte Carlo (MCMC) methods. Consistent with previous studies, the biphasic model supported the data more decisively than the single-phase model with a log Bayes factor of 35.60 (*P*<0.0001). Based on the fit to the biphasic model, the first and second decay phases were estimated to have a half-life of approximately 18.2 (17.3 to 19.2, 95%CI) and 433 (227 to 6400, 95%CI) months, respectively. We employed a 2-compartment model for our biphasic analysis. The first compartment contains quickly decaying proviruses while the second compartment is composed of more stable and slower decaying proviruses. To estimate the inflection point, we determined when the composition of the reservoir was equally composed of proviruses from the first compartment and the second compartment. The average inflection point occurred after 4.33 (2.18 to 7.54, 95%CI) years for the intact reservoir. Mathematically this inflection point is a function of the initial concentration of proviruses from the 2 compartments as well as their associated decay rates. Assuming there is minimal interaction between the 2 compartments, the inflection point is governed by the equation where A and B are the initial concentrations of proviruses in the first and second compartments respectively, and α and β are the decay rates of the first and second compartment, respectively.

One of the primary forces driving HIV reservoir persistence is clonal expansion of infected T-cells [5, 14, 26–28]. The proviral sequence data allows us to identify proviral clones and proliferation events over time. By reducing clonal sequences, we can evaluate the dynamics of the reservoir while limiting the effects due to clonal expansion. To reduce clones, we represent proviral clonal sequences once at the first time the sequence appears. Our analysis of proviral clones is limited to the largest ones, because only the largest clones are likely to be detected as repeated sequences since we sample less than one millionth of the proviruses present in the body. We then evaluated the fit of our 2 models to the new data (Figure 1B). Interestingly, when we consider the biphasic model compared to the single-phase model, the log Bayes factor drops from 35.60 with all the data included to 8.58 with clones reduced. The biphasic model is still the superior model to represent the data (*P* < 0.0001 by likelihood ratio test) with estimated half-lives of 19.8 (19.0 to 20.6, 95% CI) and 334 (190 to 2020, 95%CI) months for the 2 phases. However, because the Bayes factor has substantially decreased, this suggests that the biphasic nature of the data is weaker in the analyses with clones reduced. The average inflection point for the biphasic model fit with clones reduced occurred at 6.69 (0.57 to 12.8, 95% CI) years. Overall, our analysis suggests clonal expansion plays an important role in the biphasic nature of reservoir dynamics.

### Modeling Defective HIV Reservoir Dynamics

We repeated the analysis to measure decay of the defective reservoir. The defective proviruses were again fit to single-phase and biphasic models using Bayesian MCMC methods (Figure 2A). With a log Bayes factor of 1.94, the biphasic model again represented the dynamics slightly better than the single-phase model (*P* = 0.011). Based on the fit to the biphasic model, the first and second phases of decay were estimated to have a half-life of approximately 84.1 (80.81 and 87.36, 95% CI) and 100 (87.0 to 120, 95% CI) months, respectively. We estimated the inflection point for defective proviruses as we did for intact proviruses. Based on the best fit model parameters, this inflection point might occur after 24.7 (11.4 to 37, 95%CI) years for the defective reservoir. Additional data points at later time points are needed to validate this inflection point.

We then limited the effects of clonal proliferation by removing the definitive proviral clones (Figure 2B). The biphasic model still explained the dynamics in the data slightly better than the single-phase model based on log Bayes factors of 1.37 (*P* = 0.043). With the clones reduced, the best-fit parameters for the biphasic model yielded a first phase half-life of 79.0 (71.2 to 87.8, 95% CI) months and a second phase half-life of 146 (110 to 210, 95% CI) months, respectively. Based on the best fit model parameters, the average inflection point is predicted to occur at ~26.3 (1.31 to 54.0, 95% CI) years. In both scenarios, with all data included and with clones removed, there is a less decisive fit to the defective data between the single-phase and biphasic models when compared to the fits of the 2 models to the intact data.

Interestingly, for both data scenarios, the single-phase model had a slightly lower Akaike Information Criteria corrected for small sample size (AICc). This slight disparity likely arises because AICc only considers the maximum likelihood whereas Bayes Factor methods consider the entire posterior probability distribution for both models. Moreover, both models had similar measurements of Root Mean Squared Error (RMSE). These results can be found in Tables 1 and 2. Taken together, dynamics are predicted equally by both models for the defective reservoir [12, 29].

The striking difference in decay of defective and intact proviruses has many implications and suggests there are important differences in forces exerted on these 2 proviral forms. To investigate the source of these disparate forces, we next looked at the effect of each genomic element on dynamics among proviruses.

Akaike Information Criteria with correction (AICc) for small sample sizes and Root Mean Square Error is shown as a measure of quality of the models. The AICc and RMSE were lower for the biphasic model than the single-phase model for intact reservoir data for both scenarios, when all of the data was considered and when large clones were removed. When considering the defective reservoir data, the AICc is lower for the single-phase model in both scenarios; however, both models yield similar RMSE values.

As a measure of support between the 2 models, Bayes factors are given comparing the biphasic model to the single-phase model. For the intact reservoir data, the evidence in support of the biphasic model is decisive; however, for the defective reservoir data, there is much less evidence in support of the biphasic model.

### Genomic Element Analysis in PBMC

We wanted to determine the forces that govern reservoir dynamics with finer resolution by asking whether there were any elements within the HIV genome that correlated with decay, persistence, or clonal expansion. To do this, we designed a machine-learning approach that dissected the role of individual genetic elements in an unbiased fashion. We analyzed the frequency of every 4-element combination over time in each individual and then performed logistic regression on each combination. We chose to analyze the elements in combinations as many HIV elements are known to interact, such as splice donors and acceptors, to make functional mRNA that encode HIV proteins. As described in the methods, we calculated a force factor for each element based on the distribution of the regression rate coefficient for each combination (Figure 3). When considering all sequences, our results indicated that there was a strong relationship between the splice donor site D1 and proviral decay (*P* = < 0.001 and mean force factor = -0.847). Simultaneously, we found that HIV *nef* was the element most heavily associated with HIV persistence (*P* = 0.003 and mean force factor = 0.507).

We next analyzed proviral sequences with the large (more definitive) proviral clones eliminated (Figure 3B) by removing all repeated sequences from our analysis. We found that D1 was again the element most heavily associated with proviral decay (*P* < 0.001, mean force factors = -0.604).

Finally, we analyzed proviral clones defined as repeated sequences alone. Analysis of these definitive proviral clones provided additional insights into selection pressures (Figure 3C). D1 was again most heavily associated with proviral decay (*P* < 0.001, mean force factor = -0.897), reinforcing the strength of this correlation. Because this analysis was only performed on large clones, elements that associate with persistence may be drivers of clonal expansion. Among all elements, *tat* had the largest positive force factor suggesting *tat* may play a role in clonal proliferation (*P* = 0.038, mean force factor = 0.356). Moreover, it is striking that the *tat* element favors contraction among unique proviral sequences (when clones are removed), consistent with toxic effects of the tat protein. Given the overlapping nature of accessory genes in the 3’ end of HIV, it is remarkable that our approach disentangled the role of *tat* among the accessory genes. Moreover, there is rationale for why *tat* could enhance proviral clonal proliferation.

### Proviral Structural Effects on HIV Protein Expression

**Figure 4.**
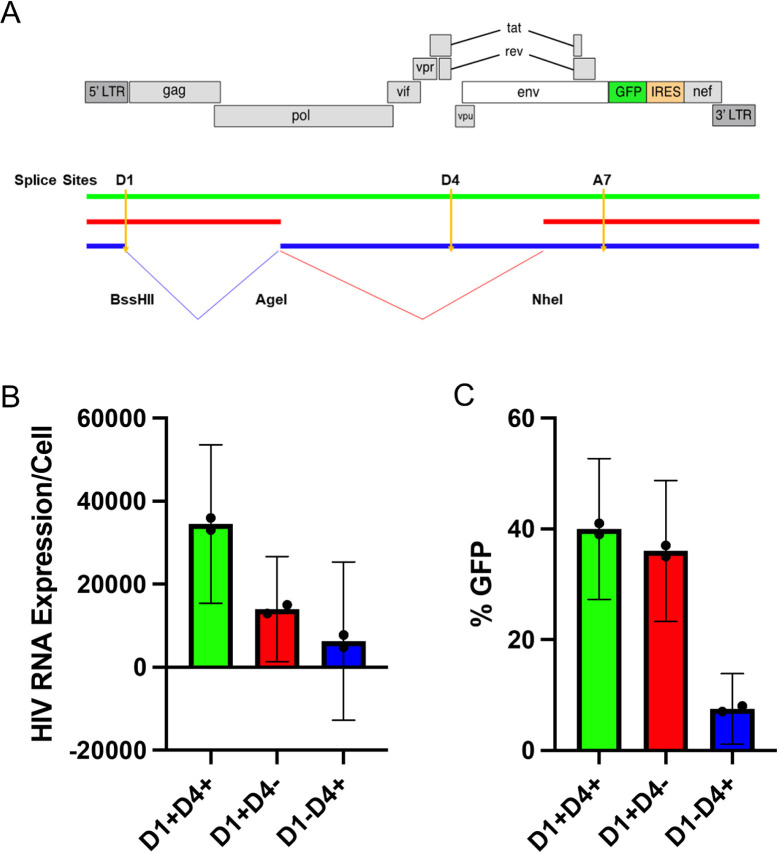
**Proviruses that contract (D1+) appear to favor protein expression.** A) The construct labeled as red represents the common 3’ deleted proviruses that contract over time while the common 5’ deleted proviruses are labeled as blue. We generated these categories by restriction enzyme digestion and regulation after filling in with Klenow Fragment. Both clones were engineered to be able to express GFP after D1 spliced to A7 (red) or after D4 spliced to A7 (blue). B) HIV RU5 RNA levels were measured by RT-PCR and (C) percentage HIV GFP expression was measured by flow cytometry. The 95% confidence intervals for (B) and (C) were derived from the t distribution. Each proviral category appear to express distinct levels of HIV RNA. Proviruses that lack D1 express significantly less HIV GFP.

The major Splice Donor site D1 is required for an intact 5’ Untranslated Region (UTR). This 5'UTR should be present in every canonical spliced and unspliced mRNA of HIV. We wanted to test if a D1 deletion results in lower HIV protein expression. Our prior work suggested that there were 3 broad categories of proviruses which exhibited differential proviral clearance after initiating ART. Those 3 proviral categories in order of clearance rate are intact proviruses (D1+D4+), 3’ deleted proviruses (D1+D4-), and 5’ deleted proviruses (D1-D4+). To test the effect of proviral structure on protein expression, we generated 3 prototype proviruses (Figure 4A) by modifying an HIV plasmid (PLENGI1) using restriction enzymes to create either a D1+, D4-, or D1-D4+ deleted provirus as well as an intact D1+D4+ provirus [30]. The PLENGI1 plasmid has GFP engineered to be in frame with *nef*. Critically, we engineered all 3 proviral prototypes to contain a functional splicing donor and acceptor as well as a complete ORF to express HIV GFP in frame with *nef*. We transfected 293 T cells with the 3 constructs and measured HIV RNA and protein expression as assessed by GFP/nef expression 24 hours later. The levels of HIV RNA appeared to be higher in the intact compared to the D1+D4-deleted proviruses which appeared slightly higher than the D1-D4+ deleted proviruses (Figure 4B, not statistically different). More importantly, HIV GFP expression levels were similar between the intact and D1+D4-deleted proviruses and dramatically higher than the D1-D4+ deleted proviruses (Figure 4C, statistically different as CI are not overlapping). Taken together, the expression levels of HIV proteins depend on proviral structure in a predictable manner. Our major conclusion is that the 5’ UTR likely enhances translation, providing a potential mechanism for why D1+ proviruses decay faster [14, 31]. It is likely this region contains genetic elements that promote translation [14, 24, 31]. Our data also suggest that this region may increase RNA stability consistent with other studies [32]. Thus, D1+D4-deleted proviruses could decay faster than D1-D4+ deleted proviruses due to the higher levels of protein expression leading to enhanced immune clearance.

## DISCUSSION

We modeled the HIV reservoir over time using near full-length HIV sequences. To gain a better understanding of the HIV reservoir dynamics, we analyzed 7 chronically infected individuals who had been sampled deeply for up to 20 years post-ART initiation. Consistent with others [6, 12, 14, 19, 33], our modeling demonstrated that the dynamics of intact HIV proviruses differed significantly from defective proviruses. To increase our resolution, we developed a machine-learning approach that relies on defective proviruses to characterize their dynamics in an unbiased fashion. Using this computational approach, we were able to dissect the contribution of each genetic element toward HIV contraction, persistence, and expansion. We found an intact 5'UTR (defined by an intact D1) was most strongly associated with decay, revealing that the 5'UTR might be important for protein expression which we bolster with experimental data. The same approach supports that *nef* is associated with persistence of HIV proviruses while *tat* is associated with clonal expansion. Thus, we provide a method to analyze longitudinal data that reveals new insights into selection pressures experienced by integrated HIV DNA. Overall, full-length sequencing provides a unique advantage compared to other reservoir measures, as we can use this information to dissect selection at the nucleotide level to determine which genetic elements drive proviral decay and expansion.

Our analysis showed that the intact reservoir decays in a distinct biphasic fashion with an inflection point from the first to second phase of decay occurring at ~4.33 years (Figure 1). Mathematically this inflection point is a function of the initial concentration of proviruses from the 2 compartments as well as their associated decay rates. Conversely, the defective reservoir did not demonstrate the same biphasic decay dynamics (Figure 2). This difference is likely due to differences in viral protein expression. Intact proviruses express all of the HIV proteins at high levels while defective proviruses express fewer proteins and at lower levels. Thus, cells harboring intact HIV are more susceptible to immune clearance as well as viral protein toxicity.

There have been several studies aimed at characterizing HIV reservoir dynamics. However, many of these studies use methods that do not capture changes in HIV nucleotide sequences over time [6, 14, 15, 19, 29, 34, 35]. In fact, there are only a few longitudinal full-length sequencing studies and these have provided important insights into selection pressures over time [14–16, 34, 36]. Importantly, our estimates of reservoir dynamics utilizing sequencing are consistent with previous reports showing that the reservoir decays in a multiphasic fashion [11, 12, 29].

Our prior reservoir analysis of contraction and expansion was biased, as we made assumptions regarding which genetic elements would likely lead to efficient protein expression, enhanced cell division, or protection from immune clearance. To overcome this bias, we divided the provirus into 33 genetic elements to analyze each element individually (Figure 3). The innovation behind the approach is that deletions of specific genetic elements provide a mechanism to dissect the forces of contraction and expansion controlled by each element through a process of elimination. While studying defective proviruses in addition to intact ones can be criticized as less relevant, they provide a unique experiment in nature to evaluate the role of individual genetic elements.

Conceptionally, we reasoned that the forces exerted on large proviral clones would be distinct from other proviral sequences. Thus, we performed a separate analysis of large clones (repeated sequences) from smaller clones (sequences detected once). Expansion forces become more apparent by analyzing the large clones separately while contraction forces become more apparent by studying the sequences detected once. We recognize that we cannot fully separate the 2 forces. Sequences that were detected once most likely represent smaller proviral clones rather than unique sequences [37]. In other words, when we separate our analysis on unique sequences compared to repeated sequences, we are likely studying selection exerted on smaller versus large proviral clones. Nonetheless, this simple approach provided clues into contraction and expansion forces.

Our analysis of HIV sequences revealed the splice donor site D1 favored contraction (Figure 3). We previously proposed a mechanism to explain the critical role of D1: specifically, every spliced and unspliced canonical HIV RNA contains the same 5’ UTR due to splicing from the major D1 donor sequence [24]. Thus, an essential function of D1 may be its ability to relocate a 5'UTR near all 9 ORFs leading to more efficient translation [14].

We also showed *nef* was associated with protection of HIV proviruses. Several groups have shown a protective effect of *nef* consistent with its ability to downregulate MHC and CD4 among other functions [38–50] protecting the provirus from clearance. Notably, Nef also has the potential to promote clonal expansion since it has also been shown to promote division and survival [51, 52].

Finally, our analysis suggested that *tat* has a role in promotion of proviral clones (Figure 3C). There are many potential mechanisms for how *tat* might increase HIV persistence. First, there is evidence that *tat* promotes HIV splicing [53] in addition to HIV transcription [54–56]. Thus, *tat* expression may also increase aberrant splicing. This in turn could drive enhanced expression of downstream host genes since viral splicing to downstream oncogenes increases oncogene expression and cell division [14, 26, 57, 58]. *Tat* could also increase cell division by upregulating host gene expression. For example, HIV *tat* has been shown to promote cell survival by upregulating BCL-2, which inhibits apoptosis in lymphocytes [59, 60]. Experimental approaches to specifically block Tat activity would provide important evidence to support or refute our findings.

While the effects of D1, *nef*, and *tat* are robustly demonstrated in our small cohort, the general-izability of these results will remain unclear until this analysis is performed in a larger cohort of individuals. Previous literature showed that deleted proviruses with D1 can exhibit faster decline than proviruses without D1 which tend to be more stable over time [12, 14, 16]. These dynamics are consistent with our experimental data showing 3’ (D1+D4-) deleted proviruses likely express HIV Gag and Pol proteins more efficiently rendering them more susceptible to immune clearance (Figure 4, red) [31]. This data also bolsters evidence that HIV proteins can be expressed at high levels without Rev (Figure 4) [61–63]. At the same time, 5’ (D1-D4+) deleted proviruses express HIV proteins less efficiently and have the potential to express HIV Nef and Tat which could shield the cell from immune clearance and may enhance splicing (Figure 4, blue) [38, 39, 41–45, 64, 65].

A limitation of our approach is that we are only studying selection of viral sequences over time. For example, our approach does not consider the orientation of the provirus in the human genome nor epigenetic factors that influence HIV expression [66–68]. In addition, our approach does not consider external forces of proviral expansion such as antigen stimulation [17] and homeostasis [18]. In fact, the presence of waxing and waning forces [28] were detected in our dataset (Supplementary Data [14]). While waxing and waning forces may obscure the role of viral elements in clonal expansion, the steady increase of proviral clones over time suggests a viral element such as *tat* may play a role in proviral clonal expansion (Figure 3C). Similarly, our approach does not distinguish if the resulting clearance is immune mediated or due to viral protein toxicity. Nonetheless, this approach is revealing because the forces of viral contraction are initiated by protein expression and so can be captured as clearance of an element.

Our computational analysis further supports the idea that opposing forces of contraction and expansion obscure a highly dynamic HIV reservoir [11, 13, 14]. We provide strong evidence that individual elements are important for reservoir decay and for protection from clearance, as well as stimulation of clonal proliferation. As such, our work supports approaches to increase D1 mediated expression and inhibit *nef* and *tat* in order to enhance reservoir decay. Additional studies to probe the role of these elements and to examine these effects in larger cohorts with greater sequencing depth are warranted. We envision machine-learning tools can be utilized to analyze how interventions perturb the not-so-stable HIV reservoir.

## SUPPLEMENTARY DATA

Supplementary materials are available at the *Pathogens and Immunity* website. Supplementary data may be provided by the authors to benefit the reader. Supplementary data are not copyedited and are the sole responsibility of the authors. Questions or comments related to supplementary materials should be addressed to the corresponding author.
