# Supplementary material for: Machine Learning Bolsters Evidence That D1, Nef, and Tat Influence HIV Reservoir Dynamics
Source: Pathog Immun. 2024 Jan 23;8(2):37–58. doi: 10.20411/pai.v8i2.621 (PMC10827039; doi:10.20411/pai.v8i2.621)
Supplement: Supplementary Tables and Figures [file pai-8-037-s01.pdf]

PATHOGENS AND IMMUNITY
IN ASSOCIATION WITH CASE WESTERN RESERVE UNIVERSITY AND THE RICHARD J. FASENMYER FOUNDATION

SUPPLEMENTARY MATERIALS

Force Factor Calculation

The order of operations for calculating force factors is as follows:

a. We first choose to examine one 4 element combination. We determined the presence or absence of each element at each time point.

b. We then perform logistic regression on the binary encoded proportion of the amount of the 4 element combination at each time point to determine the effect of each element on proviral decay or persistence via the logistic rate parameter (β_1).

$$p(x) = \frac{1}{1 + e^{-(\beta_0 - \beta_1 x)}}$$

c. We then repeated this process after choosing every 4-element combination and determining the presence and absence of all elements at each time point, followed by logistic regression.

d. Next, we prepared a table of all the element combinations and their associated slopes and rank ordered them from the smallest to the largest rate parameter.

e. We then isolated the most extreme combinations, choosing the 5% (2046 combinations) with the lowest rate parameters and the 5% with the highest rate parameters.

f. Force factors were calculated for each element by considering the number of occurrences of the element in the lower 5% and subtracting from it the number of occurrences of the element in the upper 5% then diving the result by the total number of possible occurrences (2046)

$$ForceFactor = \frac{Element_{upper} - Element_{lower}}{2046}$$

Cannon L, Fehrman S, Pinzone M, Weissman S, O'Doherty U. Machine Learning Bolsters Evidence That D1, Nef, and Tat Influence HIV Reservoir Dynamics. Pathogens and Immunity. 2024;8(2):37–58. doi: 10.20411/pai.v8i2.621

Supplemental Figure1: Force Factor Calculation

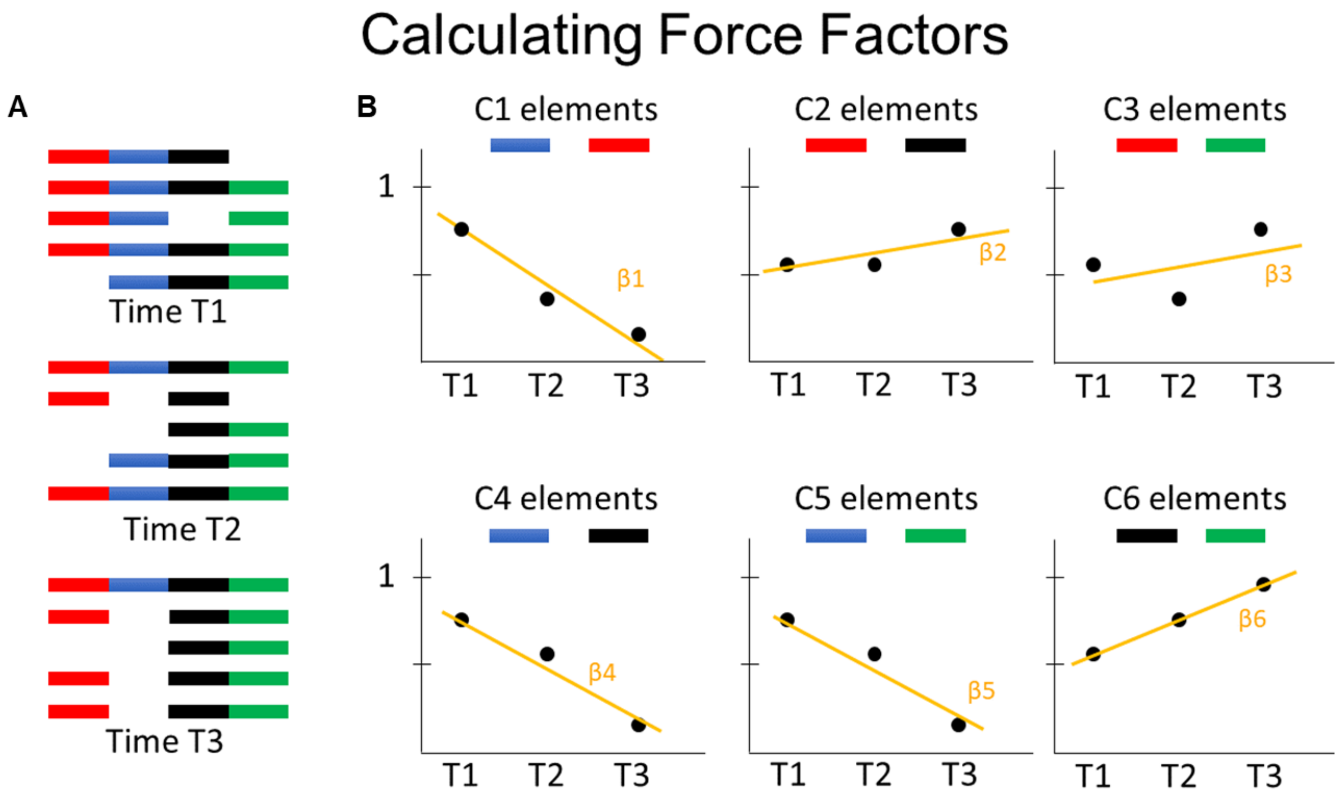

C

Combination	Element1	Element2	Slope	Order
C6	Black	Green	β6	1
C3	Red	Green	β3	2
C2	Red	Black	β2	3
C5	Blue	Green	β5	4
C4	Blue	Black	β4	5
C1	Blue	Red	β1	6

D

Element	# in upper	# in lower	Force Factor
Red	1	1	0.000
Blue	0	2	-1.000
Black	1	1	0.000
Green	2	0	1.000

Mock Experiment to Explain Force Factor Calculation.
(A) To illustrate how Force Factors are calculated we created a genome that consists of four elements (red, blue, black and green). In this mock experiment, we sequenced five genomes at each of three different time points (T1, T2 and T3). Similar to the intact and defective nature of

actual HIV genomes in vivo, our genomes can be intact, containing all four genomic elements, or they can be defective and only contain a subset of the four elements.

(B) For this mock analysis, we selected all two-element combinations of the four elements for a total of 6 possible combinations. (C1, C2, C3, C4, C5, C6). For each combination, we determined the proportion of genomes at each time point that contain the elements in the combination and then performed logistic regression to determine the rate parameter (slope). For ease of understanding, element proportions and linear slopes are shown in the figure. The elements that are associated with decay will have a negative slope and elements that are protective will be associated with a positive slope. For each combination, we calculate the corresponding slope (β1, β2, β3, β4, β5, β6). **(C)** We prepare a table of every combination with their corresponding slope and rank them from highest to lowest slope. We then focused on the combinations that had the biggest effect by studying the extremes. For this example, we consider the combinations in the lower ~30% and upper ~30% in terms of their slopes (i.e., the two combinations with the highest slopes and the two combinations with the lowest slopes). **(D)** To calculate the force factor for a given element we count the number of occurrences of that element in lower group and subtract from it the number of occurrences of that element in the upper group then divide by the total amount of combinations in one group. For example, calculation of the force factor for the blue element and green elements are:

$$Blue_{FF} = \frac{Blue_{upper} - Blue_{lower}}{Total\ Combinations\ one\ group} = \frac{(0-2)}{2} = -1$$

$$Green_{FF} = \frac{Green_{upper} - Green_{lower}}{Total\ Combinations\ one\ group} = \frac{(2-0)}{2} = 1$$

The force factor ranges from -1 to 1. The closer a force factor is to -1 the more the element is related to decay. Conversely, the closer a force factor is to 1 the more the element is related to persistence. In this simple example the blue element has a force factor of -1 and therefore would be heavily associated with decay and the green element has a force factor of 1 and is therefore associated with persistence.

PATHOGENS AND IMMUNITY
IN ASSOCIATION WITH CASE WESTERN RESERVE UNIVERSITY AND THE RICHARD J. FASENMYER FOUNDATION

Supplemental Table 1: List of the Sequences of Splice and Packaging Sites Used to Annotate the Sequenced Proviruses

***D1**	GGTRAGT
D1a	RGTAAGA
D2	GGTGAAGGGG
D3	GGTAGGA
***D4**	AGTAAGT
A1	AAATTTTCGGGTTTATTACAGG (3 mismatches allowed)
A1a	TCTTAAAATTAGC (1 mismatch allowed)
A2	ATTGTTTTTCAGA (1 mismatch allowed)
A3	ATTCATTTCAGA (1 mismatch allowed)
***A4a**	TKTGYTTCWYRAMAAAAGS
***A4b**	SCTTAGG
***A4c**	TWTCATTGCCAAGT
***A5**	GCATCTCCTATGGCAGG
***A7**	YTRTCRTTBCAGA (1 mismatch allowed)
***SL1**	ACTCGGCTTGCTGARGYGCRCWCRGCAAGAGGCGAG (4 mismatches allowed)
***SL2**	CGGCGRCTGGTGAGTACGCC (2 mismatches allowed)
***SL3**	GACTAGCGGAGGCTAG (1 mismatch allowed)
***SL4**	GGTGCGAGAGCGTC (1 mismatch allowed)
V1	TGCACTGATTTGAAGAATGATACTAATACCAATAGTAGTAG CGGGAGAATGATAATGGAGAAAGGAGAGATAAAAAAC
V2	TGCTCTTTCAATATCAGCACAAGCATAAGAGGTAAGGTGCA GAAAGAATATGCATTTTTTTATAAACTTGATATAATACCAATA GATAATGATACTACCAGCTATAAGTTGACAAGTTGT

Cannon L, Fehrman S, Pinzone M, Weissman S, O'Doherty U. Machine Learning Bolsters Evidence That D1, Nef, and Tat Influence HIV Reservoir Dynamics. Pathogens and Immunity. 2024;8(2):37–58. doi: 10.20411/pai.v8i2.621

V3	TGTACAAGACCCAACAACAATACAAGAAAAAGAATCCGTATCCAGAGAGGACCAGGGAGAGCATTTGTTACAATAGGAAAAATAGGAAATATGAGACAAGCACATTGT
V4	TGTAATTCAACACAACTGTTTAATAGTACTTGGTTTAATAGTACTTGGAGTACTGAAGGGTCAAATAACACTGAAGGAAGTGACACAATCACCCTCCCATGC
V5	TGTAATTCAACACAACTGTTTAATAGTACTTGGTTTAATAGTACTTGGAGTACTGAAGGGTCAAATAACACTGAAGGAAGTGACACAATCACCCTCCCATGC
gag	Location of ORF as defined by HXB2. Start and stop codon +/- 20 nucleotides
pol	Location of ORF as defined by HXB2. Start and stop codon +/- 20 nucleotides
vif	Location of ORF as defined by HXB2. Start and stop codon +/- 20 nucleotides
vpr	Location of ORF as defined by HXB2. Start and stop codon +/- 20 nucleotides
vpu	Location of ORF as defined by HXB2. Start and stop codon +/- 20 nucleotides
env	Location of ORF as defined by HXB2. Start and stop codon +/- 20 nucleotides
nef	Location of ORF as defined by HXB2. Start and stop codon +/- 20 nucleotides
rev	Location of ORF as defined by HXB2. Start and stop codon +/- 20 nucleotides
tat	Location of ORF as defined by HXB2. Start and stop codon +/- 20 nucleotides
***RRE**	Nucleotides 7709-8063 of HXB2 sequence (www.hiv.lanl.gov)

All elements that were required to define a provirus as intact are indicated in bold with asterisks. The Trans-activation response element was not included as we did not capture its entire sequence with our cloning strategy. We accepted both the canonical D1 sequence (GGTRAGT) as well as a GT dinucleotide cryptic donor site located four nucleotides downstream from D1.

PATHOGENS AND IMMUNITY
In Association with Case Western Reserve University and The Richard J. Fasenmyer Foundation

Criteria for Excluding Sequences:

To avoid ambiguous nucleotides due to low coverage at each end, we analyzed the region of each sequence from 20 nucleotides downstream of the 5' end primer to 20 nucleotides upstream of the 3' end primer.

On rare occasions, proviruses were excluded from analysis due to technical limitations. Specifically, proviruses were excluded based on the following:

1) Poor read coverage leading to assembly failure of consensus sequence.
2) Reads were determined to originate from more than one provirus determined by the following criteria:
    - Dinucleotide calls (>5%) within the aligned reads, suggesting more than one provirus was present during PCR amplification. Exceptions to this rule included insertions of additional adenosine nucleotides at the beginning/end of chains with at least 5 consecutive adenosine nucleotides as well as other dinucleotide calls appearing with frequency consistent with PCR error during any of the round of amplification.
    - Regions with sharp drops in coverage suggesting the presence of both a provirus with a deletion and at least one or more without a deletion.

Motifs and ORFs Identification

Sequence reads from each provirus were de novo assembled to generate a consensus sequence of each proviral genome. All possible ORFs were annotated within the assembled genomes by searching for the canonical start codon sequence ATG and extending the ORF until a stop codon was reached. The non-canonical start codon TTT was used to identify the pol gene. To be labeled as an intact HIV ORF, we required that the AUG or TTTTTT (for pol) and the stop codon to be present within 20 nucleotides of the ORF in HXB2 without premature stop codons. To identify Tat and Rev, exons 1 and 2 of Tat and Rev were annotated to the provirus genome based on 65% homology with the HXB2 Tat and Rev 1 and 2. These Tat 1/2 and Rev 1/2 homologous sequences of the provirus were then extracted, concatenated, and translated. The Tat and Rev sequences were considered intact if the sequences had no early stop codons and retained the proper stop codon. We accepted known early stops variants of Tat.

Supplemental Table 2: Clinical Characteristics

Participant	Race Sex	Age	Nadir CD4	Time Since HIV Diagnosis (Yrs)	Time on ART (Yrs)	CD4 T-Cell Count	HIV Plasma Viral Load	Antiretroviral Therapy
**CT1***	WM	47	295	24	0.9	617	<50	ATV/r/TDF/FTC
**CT2***	WM	38	0	12	0.3	386	<50	ATV/r/3Tc/d4T
**CT3***	WM	51	14	5	4.3	359	<50	AZT/3Tc/ABC
**CT4***	BF	40	194	16	1.9	287	<50	EFV/TDF/FTC
**CT5**	BM	31	114	10	3	268	<50	ATV/r/ABC/FTC
**CT6**	BM	36	241	8	5.8	470	<50	DRV/r/TDF/FTC
**CT7**	WM	59	184	UNK	18	797	<20	ATV/r/RAL

Table showing the clinical characteristics of each chronically treated (CT) study participant at the time of the first apheresis collection. Asterisks (*) denotes patients also used in Pinzone et al, 2019.

ART Abbreviations: ATV = atazanavir, r = ritonavir, TDF = tenofovir disoproxil fumarate, 3Tc = lamivudine, d4T = stavudine, AZT = zidovudine, ABC = abacavir, EFV = efavirenz, DRV = darunavir, RAL = Raltegravir

PATHOGENS AND IMMUNITY
IN ASSOCIATION WITH CASE WESTERN RESERVE UNIVERSITY AND THE RICHARD J. FASENMYER FOUNDATION

Supplemental Table 3: Intact and Defective Sequences Analyzed

Participant	Apheresis Timepoint	Intact Sequences	Defective Sequences
CT1	2008	12	84
	2010	5	135
	2012	4	99
	2015	2	126
	2018	1	122
	2019	2	125
CT2	2005	36	115
	2007	16	90
	2009	14	102
	2011	15	206
	2014	10	185
	2015	13	183
	2018	9	99
	2019	3	120
CT3	2001	17	103
	2005	53	123
	2007	4	94
CT4	2010	19	111
	2014	9	98
CT5	2015	4	97
	2016	3	87
CT6	2015	1	116
	2017	1	114
CT7	2014	4	97
	2016	1	109

Table showing the intact and defective sequences analyzed at each timepoint for each chronically treated (CT) study participant.

Cannon L, Fehrman S, Pinzone M, Weissman S, O'Doherty U. Machine Learning Bolsters Evidence That D1, Nef, and Tat Influence HIV Reservoir Dynamics. Pathogens and Immunity. 2024;8(2):37–58. doi: 10.20411/pai.v8i2.621

Supplemental Table 4: Decay Parameter Estimates

Single Phase		Intact		Defective	
Parameter	Description	All Data	Clones Reduced	All Data	Clones Reduced
γ	fixed single phase decay rate	-0.33	-0.36	-0.07	-0.09
Y01	CT1 Initial Reservoir Concentration	633	635	4116	3845
Y02	CT2 Initial Reservoir Concentration	2361	2357	4938	5009
Y03	CT3 Initial Reservoir Concentration	419	335	472	288
Y04	CT4 Initial Reservoir Concentration	2049	2162	5538	5627
Y05	CT5 Initial Reservoir Concentration	280	324	3809	3963
Y06	CT6 Initial Reservoir Concentration	146	150	2737	2466
Y07	CT7 Initial Reservoir Concentration	7320	6043	3492	3682
**Biphasic**		**Intact**		**Defective**	
Parameter	Description	All Data	Clones Reduced	All Data	Clones Reduced
α	fixed first phase decay rate	-0.46	-0.42	-0.09	-0.11
β	fixed second phase decay rate	-0.02	-0.02	-0.03	-0.06
A01	CT1 First Compartment Initial Concentration	688	632	3067	2940
B01	CT1 Second Compartment Initial Concentration	41	25	1130	914
A02	CT2 First Compartment Initial Concentration	2289	2338	4355	4523
B02	CT2 Second Compartment Initial Concentration	201	81	802	559
A03	CT3 First Compartment Initial Concentration	450	400	331	166
B03	CT3 Second Compartment Initial Concentration	45	8	95	87
A04	CT4 First Compartment Initial Concentration	1849	1876	5040	4837
B04	CT4 Second Compartment Initial Concentration	297	230	790	917
A05	CT5 First Compartment Initial Concentration	154	152	3174	3063
B05	CT5 Second Compartment Initial Concentration	93	92	838	1017
A06	CT6 First Compartment Initial Concentration	76	75	2474	1679
B06	CT6 Second Compartment Initial Concentration	11	11	541	770
A07	CT7 First Compartment Initial Concentration	150	150	3354	3332
B07	CT7 Second Compartment Initial Concentration	34	36	690	708

Cannon L, Fehrman S, Pinzone M, Weissman S, O'Doherty U. Machine Learning Bolsters Evidence That D1, Nef, and Tat Influence HIV Reservoir Dynamics. Pathogens and Immunity. 2024;8(2):37–58. doi: 10.20411/pai.v8i2.621

PATHOGENS AND IMMUNITY
IN ASSOCIATION WITH CASE WESTERN RESERVE UNIVERSITY AND THE RICHARD J. FASENMYER FOUNDATION

Table showing parameter estimates for reservoir dynamics analysis. Decay rates are given for fits for the single-phase model the and biphasic model for both cases when all data is considered and when clones are reduced.

Cannon L, Fehrman S, Pinzone M, Weissman S, O’Doherty U. Machine Learning Bolsters Evidence That D1, Nef, and Tat Influence HIV Reservoir Dynamics. Pathogens and Immunity. 2024;8(2):37–58. doi: 10.20411/pai.v8i2.621

PATHOGENS AND IMMUNITY
IN ASSOCIATION WITH CASE WESTERN RESERVE UNIVERSITY AND THE RICHARD J. FASENMYER FOUNDATION

Supplemental Table 5: *P* values and Force Factors

Element	Force Factor All	P Values All	Force Factor Clones Removed	P Values Clones Removed	Force Factor Only Clones	P Value Only Clones
**D1**	-0.847	0.000	-0.604	0.000	-0.897	0.000
**D1a**	-0.022	0.948	-0.054	1.000	0.205	0.573
**D2**	0.013	0.948	0.116	1.000	-0.027	0.984
**D3**	-0.031	0.948	-0.016	0.935	-0.022	0.922
**D4**	0.073	0.832	0.074	1.000	-0.018	0.925
**A1**	0.004	0.998	0.135	0.904	-0.012	0.937
**A1a**	-0.032	0.948	-0.028	0.938	0.066	0.573
**A2**	0.010	0.948	0.063	1.000	0.030	0.573
**A3**	0.083	0.832	0.062	1.000	-0.003	0.981
**A4a**	0.062	0.832	0.043	0.995	0.033	0.573
**A4b**	0.059	0.832	0.039	0.956	0.027	0.573
**A4c**	-0.016	0.948	0.337	0.053	-0.030	0.573
**A5**	0.056	0.958	0.030	0.962	0.027	0.953
**A7**	0.049	0.948	-0.003	0.977	0.022	0.949
**SL1**	-0.061	0.832	-0.091	1.000	-0.117	0.573
**SL2**	-0.207	0.739	-0.309	0.059	-0.122	0.573
**SL3**	-0.080	0.832	-0.072	1.000	-0.125	0.573
**SL4**	-0.083	0.832	-0.044	0.953	-0.091	0.573
**V1**	0.116	0.832	0.048	1.000	0.043	0.573
**V2**	0.089	0.832	0.061	1.000	0.045	0.573
**V3**	0.015	0.948	0.045	1.000	-0.036	0.573
**V4**	0.062	0.832	0.063	1.000	0.051	0.573
**V5**	0.062	0.832	0.065	1.000	0.051	0.573
gag	-0.169	0.739	-0.116	0.904	-0.083	0.573
pol	-0.161	0.832	-0.121	0.904	-0.073	0.573
vif	0.009	0.990	0.066	1.000	0.066	0.573
vpr	0.058	0.987	-0.018	0.952	0.045	0.573
vpu	0.055	0.928	-0.006	0.984	0.067	0.573
env	-0.001	0.994	0.167	0.904	0.294	0.105
nef	0.507	0.003	0.209	0.516	0.119	0.573
rev	0.162	0.986	-0.031	0.991	0.067	0.573
tat	0.100	0.832	-0.135	0.904	0.356	0.038
**rre**	0.066	0.832	0.026	0.925	0.040	0.573

Cannon L, Fehrman S, Pinzone M, Weissman S, O’Doherty U. Machine Learning Bolsters Evidence That D1, Nef, and Tat Influence HIV Reservoir Dynamics. Pathogens and Immunity. 2024;8(2):37–58. doi: 10.20411/pai.v8i2.621

PATHOGENS AND IMMUNITY
IN ASSOCIATION WITH CASE WESTERN RESERVE UNIVERSITY AND THE RICHARD J. FASENMYER FOUNDATION

Table showing the p values and mean force factors for each element when all data is utilized, clones are removed, and only the large clones are considered.

Significant Elements

Supplemental Figure 2: Force Factor Null Distributions

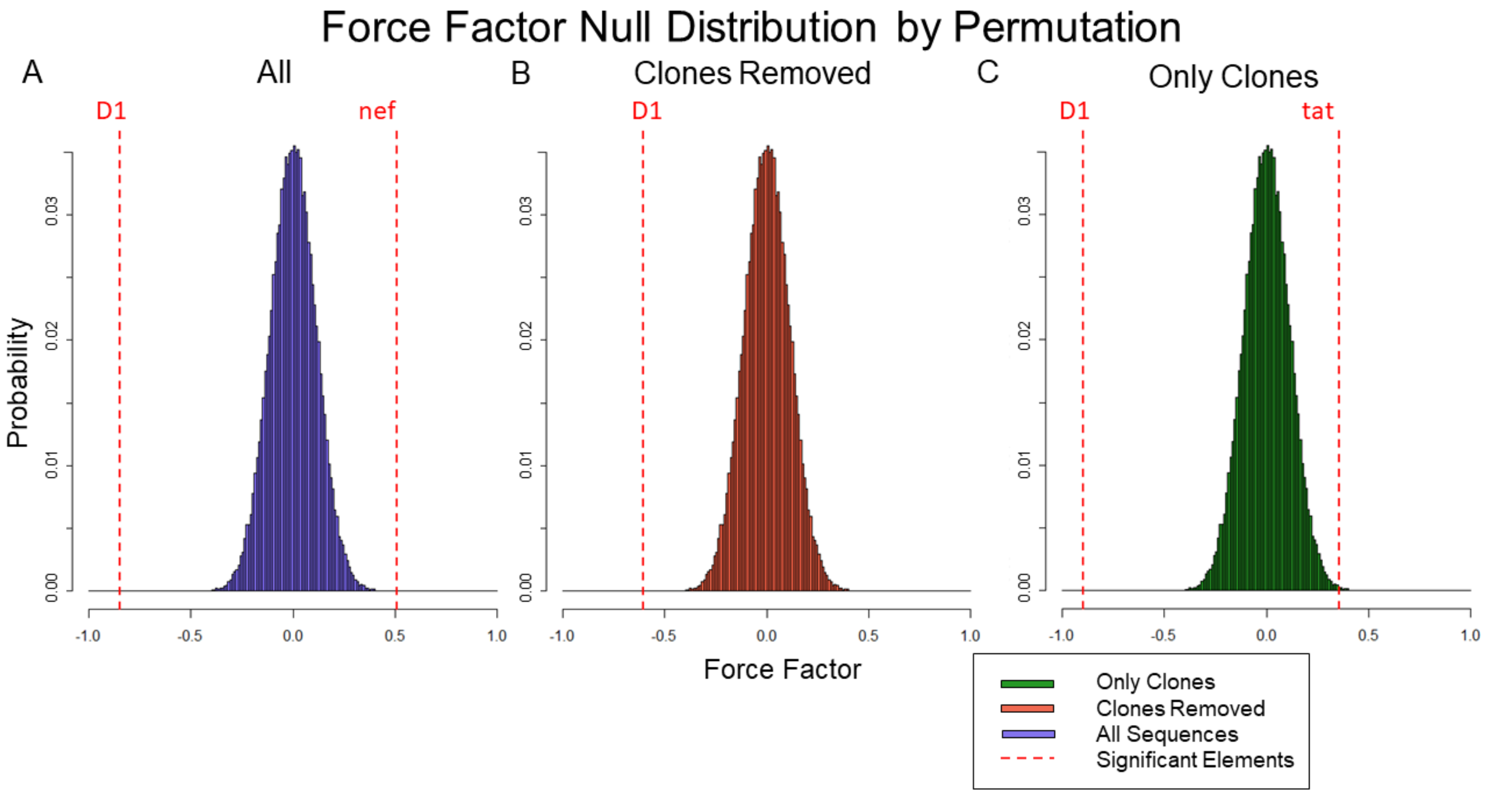

The *P* values were calculated by randomly permuting the elements in each provirus at each time point and calculating the resulting force factors. The null distribution is shown in each plot with the significant elements at their respective force factors for A) when all proviruses are considered, B) the clonal proviruses are removed, and C) when only the clonal proviruses are considered.

Cannon L, Fehrman S, Pinzone M, Weissman S, O'Doherty U. Machine Learning Bolsters Evidence That D1, Nef, and Tat Influence HIV Reservoir Dynamics. Pathogens and Immunity. 2024;8(2):37–58. doi: 10.20411/pai.v8i2.621
